# The causal association between immune cells and gout: A bidirectional two-sample Mendelian randomization study

**DOI:** 10.1097/MD.0000000000040064

**Published:** 2024-10-18

**Authors:** Yuanyuan Zeng, Mu Huang, Wen Zeng, Ling Lei

**Affiliations:** a Department of Rheumatology and Immunology, Yulin First People’s Hospital, Yulin, Guangxi Zhuang Autonomous Region, China; b Department of Rheumatology and Immunology, The First Affiliated Hospital of Guangxi Medical University, Nanning, Guangxi Zhuang Autonomous Region, China.

**Keywords:** causal relationship, gout, immune cells, Mendelian randomization

## Abstract

Gout, a metabolic disorder, is increasingly being linked to immune cells. However, the causal relationships between these factors remain unclear. Our study aimed to elucidate the causal relationship between immune cells and gout. Our study used 2-sample Mendelian randomization (MR) to explore the causal relationship between immune cells and gout. It is noteworthy that we utilized 5 methods MR-Egger, weighted median, inverse variance weighted, weighted mode, and simple mode to ensure the reliability of the results. Comprehensive sensitivity analyses were performed to verify the robustness, heterogeneity, and horizontal pleiotropy of the results. After false discovery rate correction (*P*_FDR_ <0.20), 3 immunophenotypes were identified: one in the B cell panel, one in the regulatory T cells panel, and another in the T lymphocytes, B lymphocytes, Natural Killer cells panel. Among them, 2 immunophenotypes (CD4-CD8-T cell absolute count and CD25 on IgD + CD24 + B cell) increased the risk of developing gout, whereas the other one immunophenotype (CD45RA + CD28- CD8 + T cell %T cell) decreased the risk of gout. Subsequently, we did not observe heterogeneity and horizontal pleiotropy stable in these data through comprehensive sensitivity analyses. Furthermore, we identified some positive results in reverse MR analysis, but after false discovery rate correction (*P*_FDR_ <0.20), no significant results were detected. Our study revealed causal relationships between immune cells and gout, providing novel insights into the prevention and treatment of gout.

## 1. Introduction

Gout is an arthritis characterized by the deposition of monosodium urate (MSU) crystals in joints, directly associated with hyperuricemia, leading to joint damage and significant organ impairment in vital organs such as the heart and kidneys. Epidemiological data from 2020 indicates a rising global prevalence of gout, with a notable increase in incidence with advancing age.^[1]^ The prevalence of hyperuricemia ranges from 2.6% to 36.0% in different racial populations, while the prevalence of gout ranges from 0.03% to 15.30%. In recent years, both have shown a significant increase and trend toward younger age groups.^[1]^ Hyperuricemia and chronic inflammation play important roles in gout and should be assessed prominently in all gout patients. Gout is a systemic disease affecting multiple systems, and both acute and chronic forms reduce patients’ productivity and quality of life, leading to significant socioeconomic burdens.^[2]^ Therefore, early and proactive prevention, timely diagnosis, and management of complications can significantly reduce the incidence and improve the prognosis.

Gout is an inflammatory response characterized by the deposition of MSU crystals within the joints. This process leads to the release of nucleotide-binding oligomerization domain-like receptor thermal protein domain–associated protein 3 (NLRP3), which mediate the production of interleukin (IL)-1β and other proinflammatory factors.^[3]^ Recently, an increasing number of genes and immune factors have been described as being associated with gout inflammation. These include geranylgeranyl transferase I beta subunit (PGGT1B),^[4]^ histone H3 protein (H3K4me1), IL-1β,^[5]^ interleukin-23 receptor (IL-23R),^[6]^ all of which may increase the risk of gout occurrence. Gout involves both innate and adaptive immunity. The overactivation of immune cells and the release of cytokines exacerbate tissue damage and pain, constituting one of the key mechanisms underlying gout attacks.^[7]^

The study found elevated levels of IL-17 and IL-22 in the peripheral blood of gout patients, suggesting the involvement of Th1 and Th17 immune cells in the pathogenesis of gout.^[8,9]^ Macrophages are the first immune cells to encounter MSU crystals, activating Th2 and mast cells through the serum stimulation-2 (ST2)/IL-33 pathway, thus promoting the inflammatory response.^[10]^ Neutrophil proteinase 3 (PR3) has the function of converting pro-IL-1β into IL-1β,^[11]^ neutrophil recruitment enhances the phagocytosis of MSU crystals and leukocyte chemotaxis, sustaining the inflammatory response.^[12]^ Based on single-cell RNA sequencing and independent validation cohorts, the potential mechanism underlying gout flares may involve upregulation of HLA-DQA1 + non-classical monocytes, which are associated with antigen processing and presentation.^[13]^ T cells, neutrophils, macrophages, monocytes, and cytokines such as IL-23 all participate in the development and pathogenesis of gout. Additionally, other research suggests that drugs targeting specific molecular pathways may achieve favorable therapeutic outcomes in patients with inadequate response to conventional pharmacotherapy.^[14,15]^

Compared with traditional observational studies, MR is an innovative epidemiological analysis technique that utilizes single nucleotide polymorphisms (SNPs) as instrumental variables (IVs) to assess causal relationships between exposure factors and diseases. Because genetic variations are randomly allocated at conception and conform to the temporal sequence, MR effectively addresses issues of confounding factors and reverse causality.^[16]^ In observational studies, a close association between immune cells and gout has been observed; however, the causal relationship between them remains inconsistent. Therefore, we conducted extensive bidirectional 2-sample and multivariable MR analysis to elucidate the causal relationship between immune cell characteristics and gout.

## 2. Materials and methods

### 2.1. Study design

**Figure 1.**
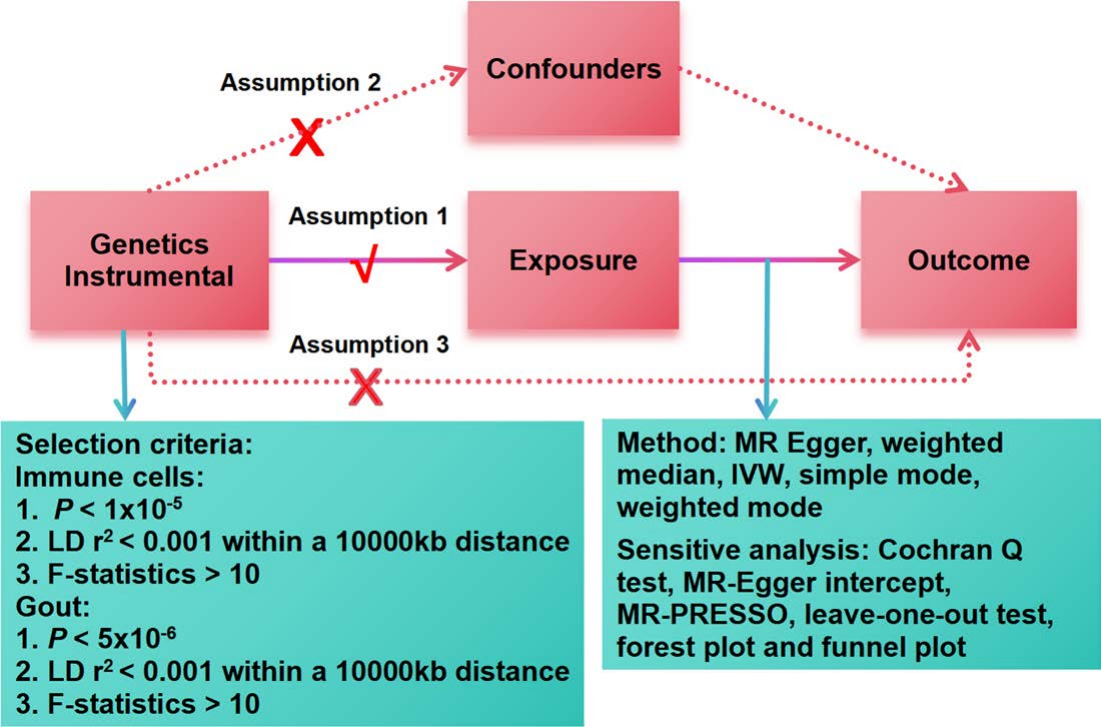
Diagram of the MR analysis. Assumption 1, genetic variants are closely related to the exposure; Assumption 2, genetic variants are not associated with potential confounders; Assumption 3, genetic variants affect outcomes only through the exposure pathway. IVW = inverse variance weighted, LD = linkage disequilibrium, MR = Mendelian randomization.

Our study investigated causal relationships between 731 immune cells and gout based on MR analysis (Fig. 1). In the process of our research, MR analysis must adhere to 3 core assumptions to ensure unbiased causal effects: genetic variants are closely related to the exposure, genetic variants are not associated with potential confounders, and genetic variants affect outcomes only through the exposure pathway.^[17]^ Our study was a secondary analysis based on publicly available data and therefore did not require ethical approval.

### 2.2. GWAS data source for immunophenotypes

The GWAS data for 731 immunophenotypes were obtained from the GWAS Catalog (GCST90001391-GCST90002121), derived from a comprehensive study of the genetic features of immune cells. The primary objective of this study was to investigate the impact of 22 million genetic variations on 731 immune cell traits using a cohort of 3757 Sardinian individuals. Additionally, the study aimed to elucidate associations between autoimmune diseases and immunological traits. The GWAS data encompassed various types, including 389 median fluorescence intensity values reflecting surface antigen levels, 192 relative cell counts, 118 absolute cell counts, and 32 morphological characteristics.^[18]^

### 2.3. GWAS data source for gout

The GWAS statistics for gout were sourced from FinnGen dataset (https://gwas.mrcieu.ac.uk/datasets/finn-b-M13_GOUT/) to conduct MR analysis, including 16,380,152 SNPs, and GWAS was done on 150,797 Europeans (nCase = 3576, nControl = 147,221).

### 2.4. IV selection

The recent research indicated that we could select important SNPs for various immune traits using a relaxed cutoff value of *P* < 1 × 10^−5^.^[19]^ The linkage disequilibrium *r*^2^ threshold was set to be <0.001 within a 10,000 kb distance to eliminate the effects of genetic linkage imbalance. Then, we select important SNPs for gout using a value of *P* < 5 × 10^−6^ and *r*^2^ <0.001 within a 10,000 kb distance. All IVs with low *F* statistics (<10) were removed to mitigate weak instrumental bias. Finally, we exclude all SNPs associated with confounding variables through GWAS Catalog dataset.

### 2.5. Statistical analysis

Our study primarily employed the inverse variance weighted method to observe the causal relationship between 731 immunophenotypes and gout, supplemented by four other methods, MR-Egger, weighted median, weighted mode, and simple mode. We comprehensively analyzed the results of these 5 methods to ensure the reliability of our findings. Subsequently, we employed false discovery rate (FDR) correction to further adjust for multiple comparisons. According to previous studies, *P*_FDR_ <0.20 was considered suggestive of a causal relationship, while *P*_FDR_ <0.05 was considered to indicate a significant causal relationship.^[20]^

Additionally, multiple methods were used to assess robustness, pleiotropy, and heterogeneity in the results. Cochran’s *Q* test was utilized to evaluate heterogeneity. MR-Egger intercept was used to address and adjust for pleiotropy. Leave-one-out test, forest plot, and funnel plot were employed to assess the robustness. MR-PRESSO test was utilized to detect horizontal pleiotropy and identify bias values. *P* < .05 was considered to indicate statistically significant. In our study, all statistical analyses were conducted using the R statistical software (version 4.3.3) with the “TwoSampleMR” (version 0.5.11).

## 3. Results

### 3.1. Causal effect from immune cells on gout

**Figure 2.**
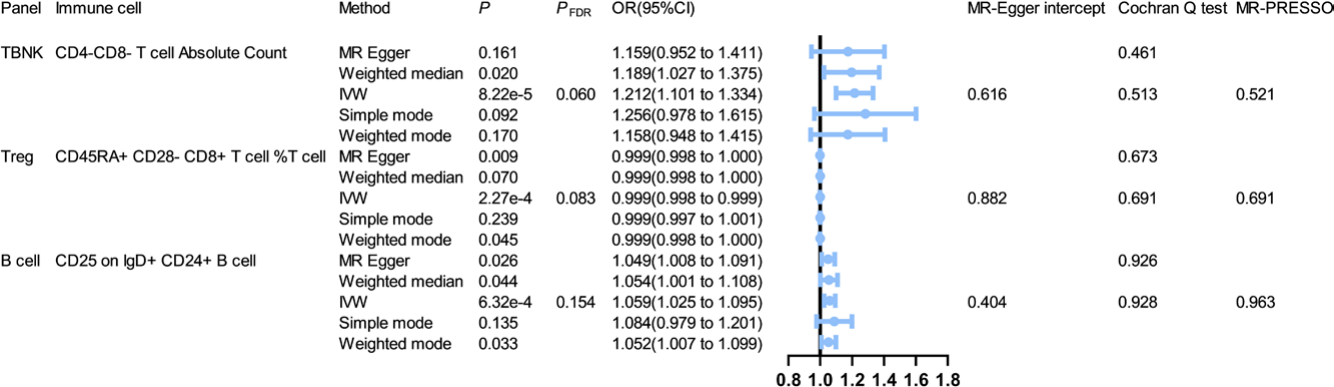
Forest plot of MR analysis. Forest plot to visualize the causal effect of immune cells on the risk of gout by Inverse variance weighted, MR-Egger, weighted median, and weighted mode method. This plot showed CD4-CD8- T cell absolute count CD25 on IgD + CD24 + B cell and may increase the risk of gout, but CD45RA + CD28- CD8 + T cell %T cell may decrease the risk of gout. CI = confidence interval, IVW = inverse variance weighted, MR = Mendelian randomization, OR = odds ratio, TBNK = T lymphocytes, B lymphocytes, and Natural Killer cells, Treg = regulatory T cells.

In our study, we first analyzed the causal effects of 731 immune cell phenotypes as exposure variables on gout (Fig. 2). After FDR correction (*P*_FDR_ <0.20), 3 immunophenotypes were identified, one in the B cell panel, one in the regulatory T cells (Treg) panel, and another in the T lymphocytes, B lymphocytes, Natural Killer cells panel. The result of inverse variance weighted method indicated that CD4-CD8- T cell absolute count (odds ratio [OR] = 1.212, 95% confidence interval [CI] = 1.101–1.334, *P *= 8.22e-5, *P*_FDR_ = 0.060) increased the risk of gout, and the other 4 methods also indicated it was a risk factor. CD25 on IgD + CD24 + B cell (OR = 1.059, 95% CI = 1.025–1.095, *P *= 6.32e-4, *P*_FDR_ = 0.154) increased the risk of gout, and the other 4 methods also indicated it was a risk factor. In addition, CD45RA + CD28- CD8 + T cell %T cell (OR = 0.999, 95% CI = 0.998–0.999, *P *= 2.27e-4, *P*_FDR_ = 0.083) reduced the risk of gout, and the other 4 methods also indicated it was a protective factor.

Furthermore, various methods were used to observe pleiotropy and heterogeneity of the results. We employed Cochran’s *Q* test to assess heterogeneity and MR-Egger intercept to account for pleiotropy, and *P* values of all results were greater than 0.05 (Fig. 2). Leave-one-out test, forest plots, and funnel plots showed that these data were very stable (Supplemental Digital Content, http://links.lww.com/MD/N716). At last, we did not observe any horizontal pleiotropy and bias values through MR-PRESSO test (Fig. 2).

### 3.2. Reverse causal effect from immune cells on gout

In reverse MR analysis, we identified some positive results, but after FDR correction (*P*_FDR_ < 0.20), no meaningful results were detected.

## 4. Discussion

Based on our study, an increased CD25 on IgD + CD24 + B cell and CD4-CD8- T cell absolute count are associated with a higher risk of gout occurrence. Various T lymphocytes are involved in gout, with Th17, Th1, Th9, Th22, and γδ T cells promoting gout attacks, while Tregs and Th2 cells exert inhibitory effects.^[21]^ Currently, research on T lymphocytes mainly focuses on the balance between Th1/Th2 and Th17/Treg cells within CD4 + T cell subtypes. Two Chinese scholars’ studies on the number of Th1 cells in the peripheral blood of gout patients yielded inconsistent results,^[8,22]^ possibly due to the small sample size included in the studies. Gout patients exhibit a natural enrichment of CD4 T cell subsets, with Th1 and Th17 immune cells confirmed to be involved in the onset of gout. The main mechanism is their overactivation leading to increased synthesis of IL-12 and IL-23, which have also become targets for immunotherapy of gouty arthritis.^[23,24]^ CD4-CD8- T cell absolute count (double-negative T cells [DNT]) are T cells positive for CD3 but lacking CD4 and CD8 coreceptors. They can express TCRαβ or TCRγδ but not natural killer T cell markers.^[25]^ Studies have shown that DNT cells originate from a specific stage of thymic cells, similar to CD4+ and CD8+ T cells. Although DNT cells constitute only 3% to 5% of peripheral blood T lymphocytes,^[26]^ unlike traditional CD4+ and CD8+ T cells, DNT cells possess both innate and adaptive immune functions. DNT cells can secrete IL-17 and interferon-γ, participating in the pathogenesis of renal damage in patients with systemic lupus erythematosus (SLE).^[27]^ Because DNT cells can migrate from peripheral blood to organs, infiltrating salivary glands, skin, and kidneys, they play a central role in the pathogenesis of Sjögren’s syndrome and psoriasis.^[28,29]^ Furthermore, our study found that DNT cells are a risk factor for gout, providing clues and potential mechanisms for our research findings based on observational studies. Additionally, we have identified CD25 on IgD CD24 B cells as a risk factor for the development of gout. Studies have found that CD25 B cells are capable of effective antigen presentation and exhibit a more mature phenotype.^[30]^ Recent research has discovered that an imbalance between CD25hi Bregs and CD4 Tregs is involved in the pathogenesis of Type 1 diabetes.^[31]^ Unfortunately, there is currently a lack of research on the relationship between gout and CD25 on IgD CD24 B cells, with no definitive findings, necessitating further investigation to confirm.

CD45RA + CD28- CD8 + T cell %T cell is a protective factor for the occurrence of gout. It is located within the Treg cell subset, which is crucial for maintaining immune tolerance and preventing autoimmunity. CD45 is a highly conserved receptor protein tyrosine phosphatase expressed specifically on all nucleated cells, regulating innate and adaptive immunity. CD45 plays a critical role in initiating T cell receptor signaling by regulating the activation of Src family protein tyrosine kinases Lck and Fyn.^[32]^ With advancing age, the number of CD45RA + T cells gradually decreases, while the number of CD45RO + T cells increases.^[33]^ CD28 is a co-stimulatory receptor expressed in most CD4 + T cells, and its loss is associated with cellular senescence^[34]^ and prolonged antigen exposure,^[35]^ leading to an increase in CD8 + CD28- T lymphocytes in chronic inflammatory processes and in elderly individuals. Studies have found a significant decrease in CD8 + CD45RA + CCR7-CD28- subsets in lupus nephritis patients compared to healthy individuals, with a shift toward the CD8 + CD45RA-CCR7 + subset.^[36]^ In active SLE patients, the expression of IL-6 and CTLA-4 is lower in the CD8 + CD28- T cell subset, suggesting a potential role for CD8 + CD28- T cells in non-active SLE.^[37]^ CD8+ Treg effector lymphocytes have been found to suppress the production of autoantibodies in SLE,^[36]^ directly correlating with the restoration of CD8 + FoxP3 + Treg lymphocyte subsets.^[38]^ CD39 is an ectonucleotidase that hydrolyzes adenosine triphosphate into adenosine monophosphate in a rate-limiting step, which is further hydrolyzed into adenosine by CD73.^[39]^ Additionally, adenosine binding to its A2A receptor expressed on T cells leads to an elevation of intracellular cyclic adenosine monophosphate levels and suppression of effector T cell function.^[40]^ Based on these studies, new insights into the interaction between immune cells and the inflammatory environment may have a significant impact on the future direction of gout drug development.

Our 2-sample, bidirectional MR analysis utilized the largest sample size GWAS dataset to comprehensively explore the causal relationships between 731 immune cells and gout. Additionally, we employed various methods to validate the stability of our results, thus our findings are deemed reliable. However, our study has some limitations. First, the summary data from the included GWAS are limited to individuals of European ancestry, which restricts the generalizability of our findings to other populations with different genetic backgrounds, as ethnicity is one of the conditions for causality. Second, the current GWAS data lack individual patient information, preventing further subgroup analysis of the current gout patient population for more precise results. Third, although our study conducted bidirectional, multivariable MR analysis, there are numerous interactions between immune cell phenotypes. A more comprehensive understanding of the relationship between gout and immune features could be achieved through further extensive basic research on immune cells.

In conclusion, our study provides theoretical support for the causal relationship between immune cells and gout, identifying the significant role of T lymphocytes in the onset of gout, thereby offering new insights for the prevention and treatment of gout.

## Acknowledgments

This study would like to thank The FinnGen database (https://gwas.mrcieu.ac.uk/datasets/finn-b-M13_GOUT/) and public catalog (GCST90001391-GCST90002121) GWAS data provided.

## Author contributions

**Project administration:** Yuanyuan Zeng.

**Supervision:** Yuanyuan Zeng, Wen Zeng, Ling Lei.

**Writing – original draft:** Yuanyuan Zeng, Mu Huang.

**Writing – review & editing:** Yuanyuan Zeng, Wen Zeng, Ling Lei.

**Data curation:** Mu Huang.

**Formal analysis:** Mu Huang.

**Methodology:** Mu Huang.

**Visualization:** Wen Zeng.

**Funding acquisition:** Ling Lei.

## Supplementary Material


