# Supplementary material for: The causal association between immune cells and gout: A bidirectional two-sample Mendelian randomization study
Source: Medicine (Baltimore). 2024 Oct 18;103(42):e40064. doi: 10.1097/MD.0000000000040064 (PMC11495775; doi:10.1097/MD.0000000000040064)
Supplement: Supplementary file 1 [file medi-103-e40064-s001.pdf]

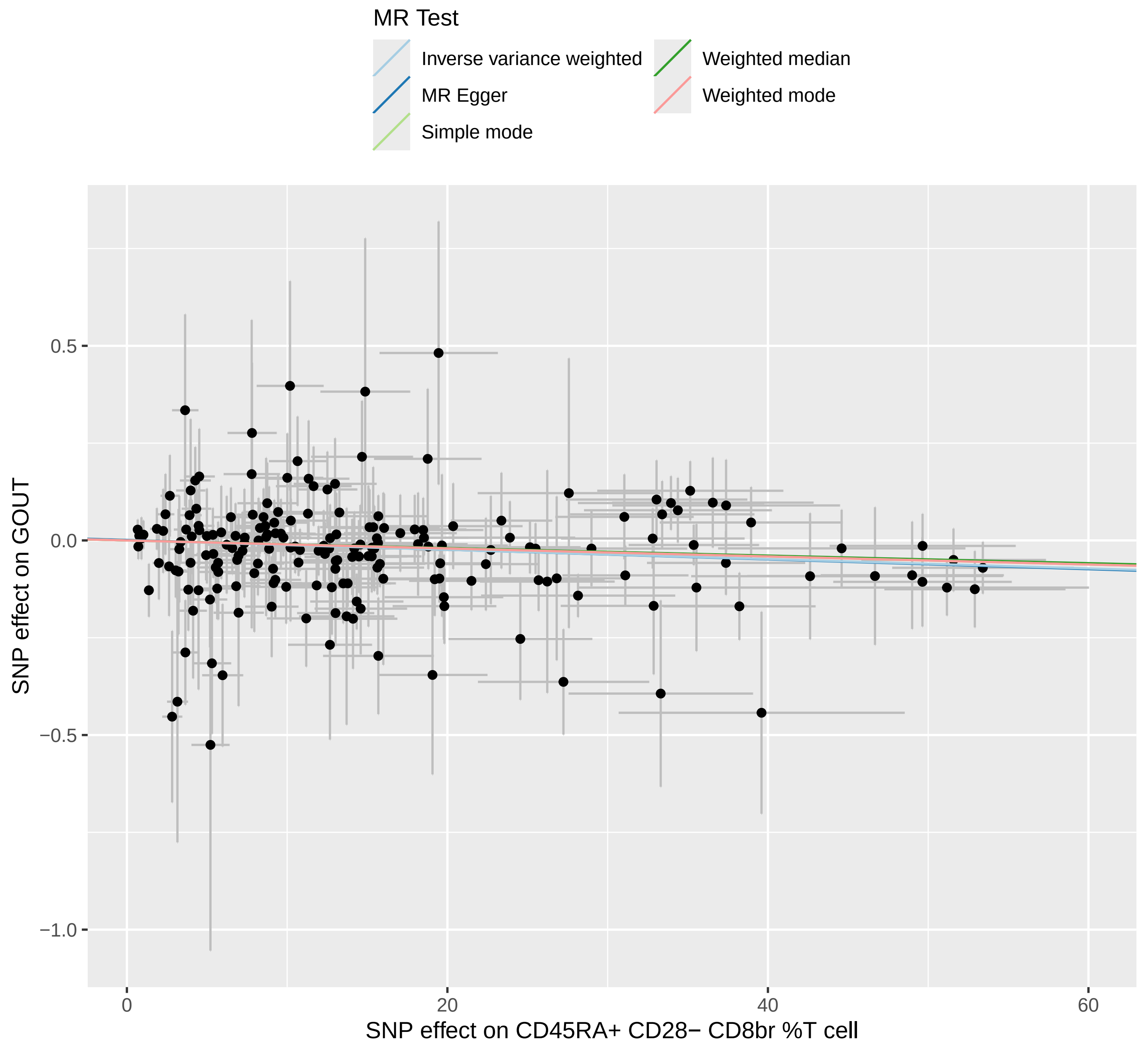
MR Test
Inverse variance weighted
MR Egger
Simple mode
Weighted median
Weighted mode
SNP effect on GOUT
0.5
0.0
−0.5
−1.0
0
20
40
60
SNP effect on CD45RA+ CD28− CD8br %T cell

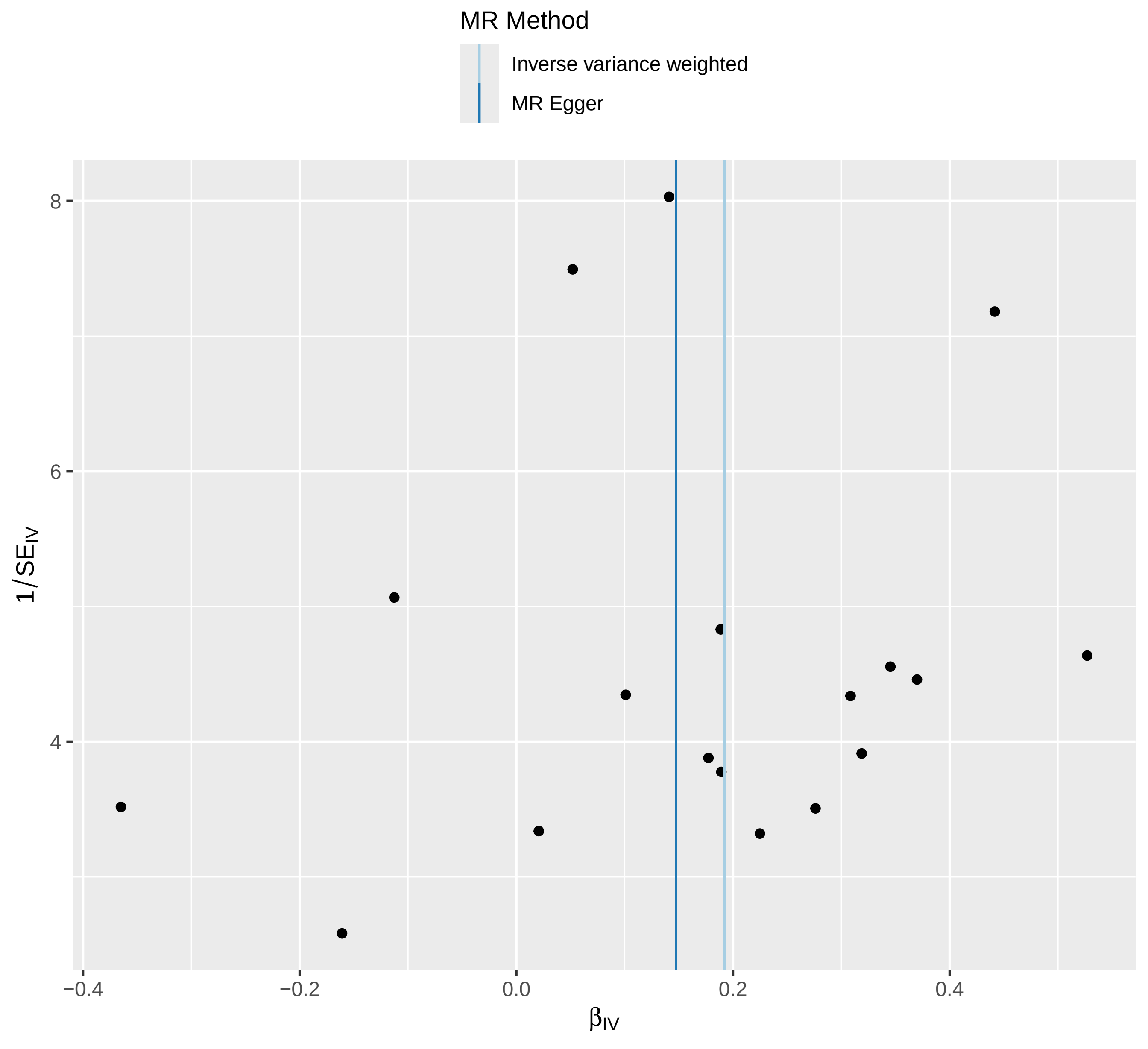
MR Method
Inverse variance weighted
MR Egger
8
6
4
1/SE$_{IV}$
−0.4
−0.2
0.0
0.2
0.4
β$_{IV}$

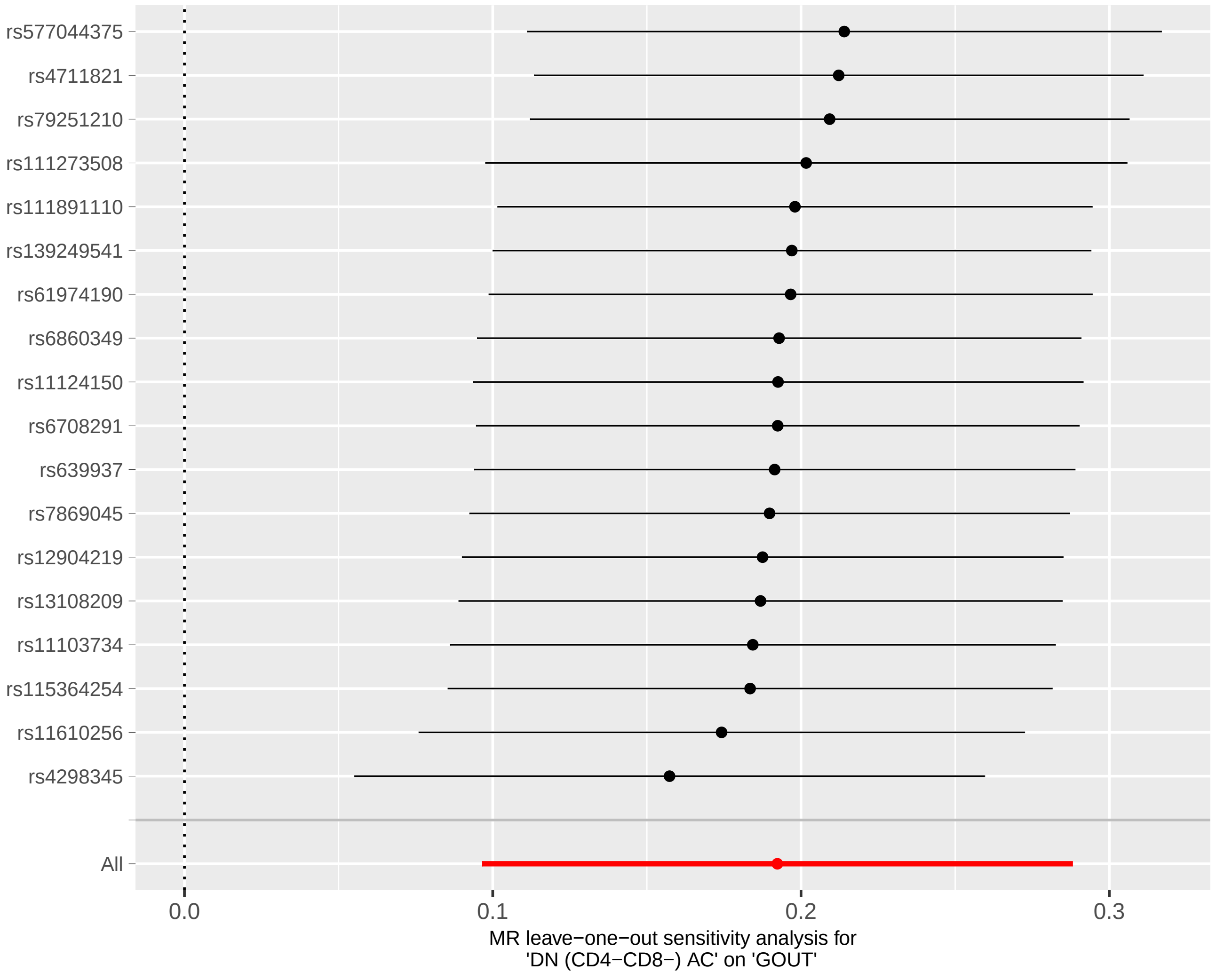
rs577044375
rs4711821
rs79251210
rs111273508
rs111891110
rs139249541
rs61974190
rs6860349
rs11124150
rs6708291
rs639937
rs7869045
rs12904219
rs13108209
rs11103734
rs115364254
rs11610256
rs4298345
All
0.0
0.1
0.2
0.3
MR leave−one−out sensitivity analysis for
'DN (CD4−CD8−) AC' on 'GOUT'

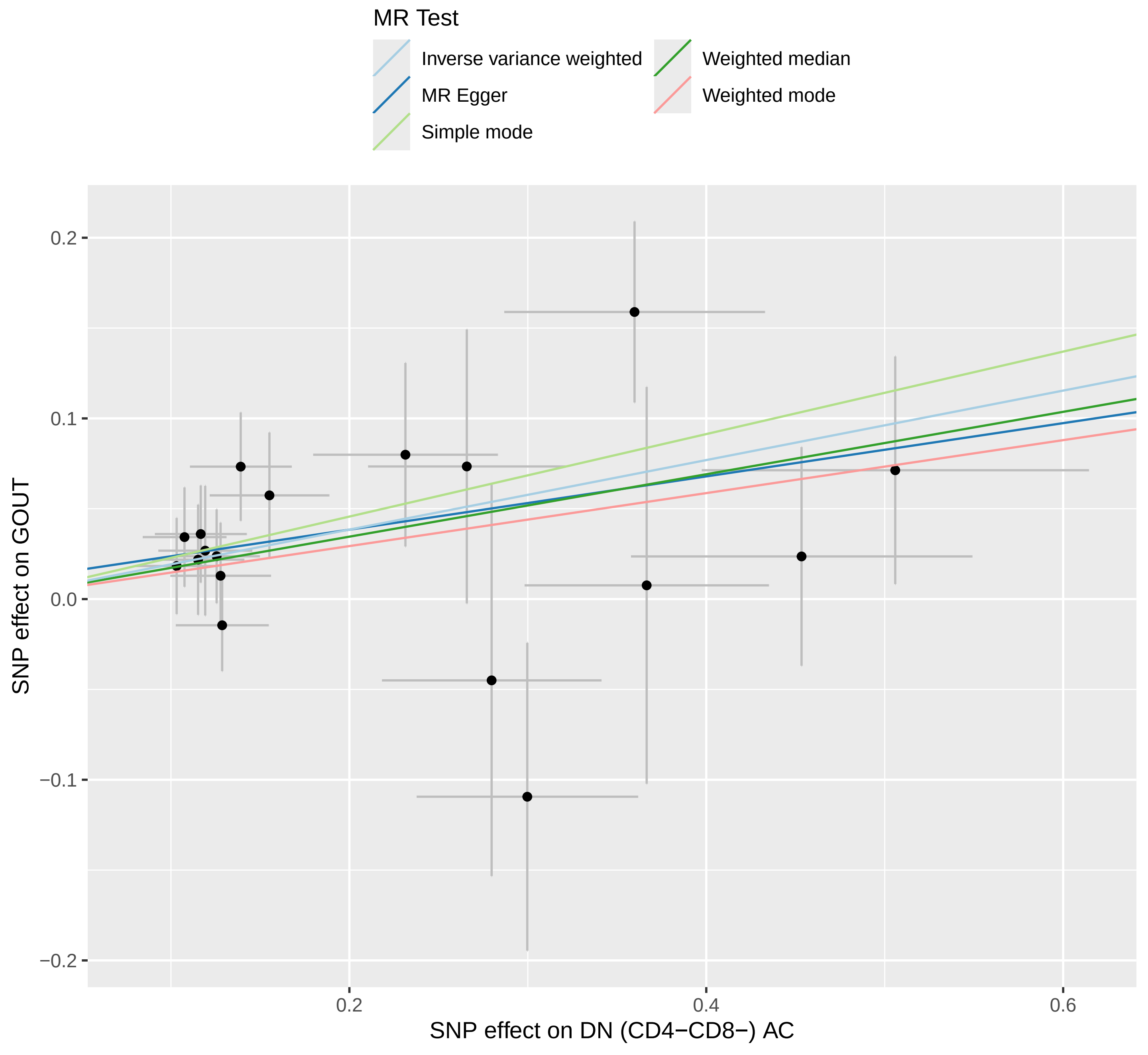
MR Test
Inverse variance weighted
MR Egger
Simple mode
Weighted median
Weighted mode
SNP effect on GOUT
SNP effect on DN (CD4−CD8−) AC
0.2
0.1
0.0
−0.1
−0.2
0.2
0.4
0.6

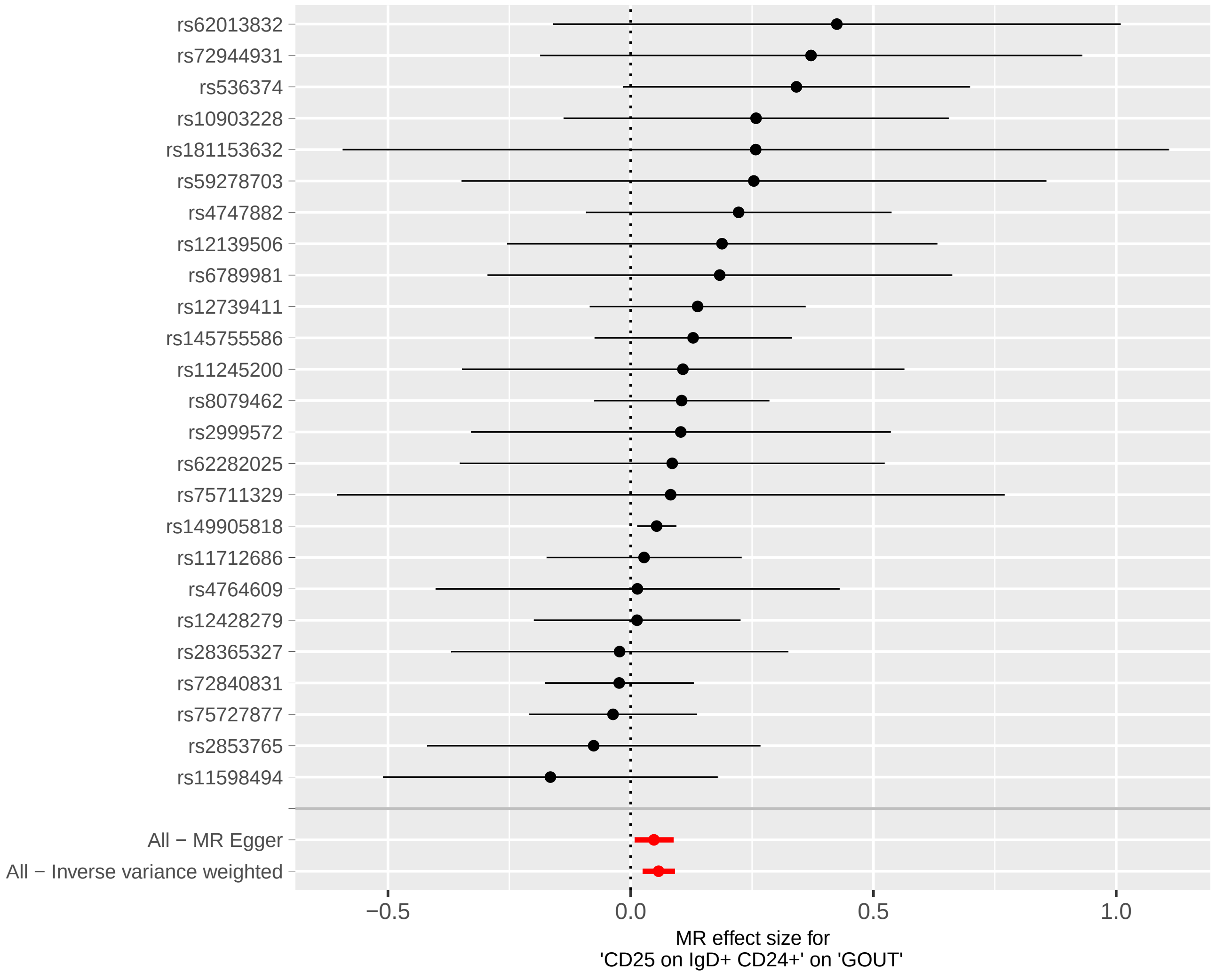

rs62013832
rs72944931
rs536374
rs10903228
rs181153632
rs59278703
rs4747882
rs12139506
rs6789981
rs12739411
rs145755586
rs11245200
rs8079462
rs2999572
rs62282025
rs75711329
rs149905818
rs11712686
rs4764609
rs12428279
rs28365327
rs72840831
rs75727877
rs2853765
rs11598494
All − MR Egger
All − Inverse variance weighted
−0.5
0.0
0.5
1.0
MR effect size for
'CD25 on IgD+ CD24+' on 'GOUT'

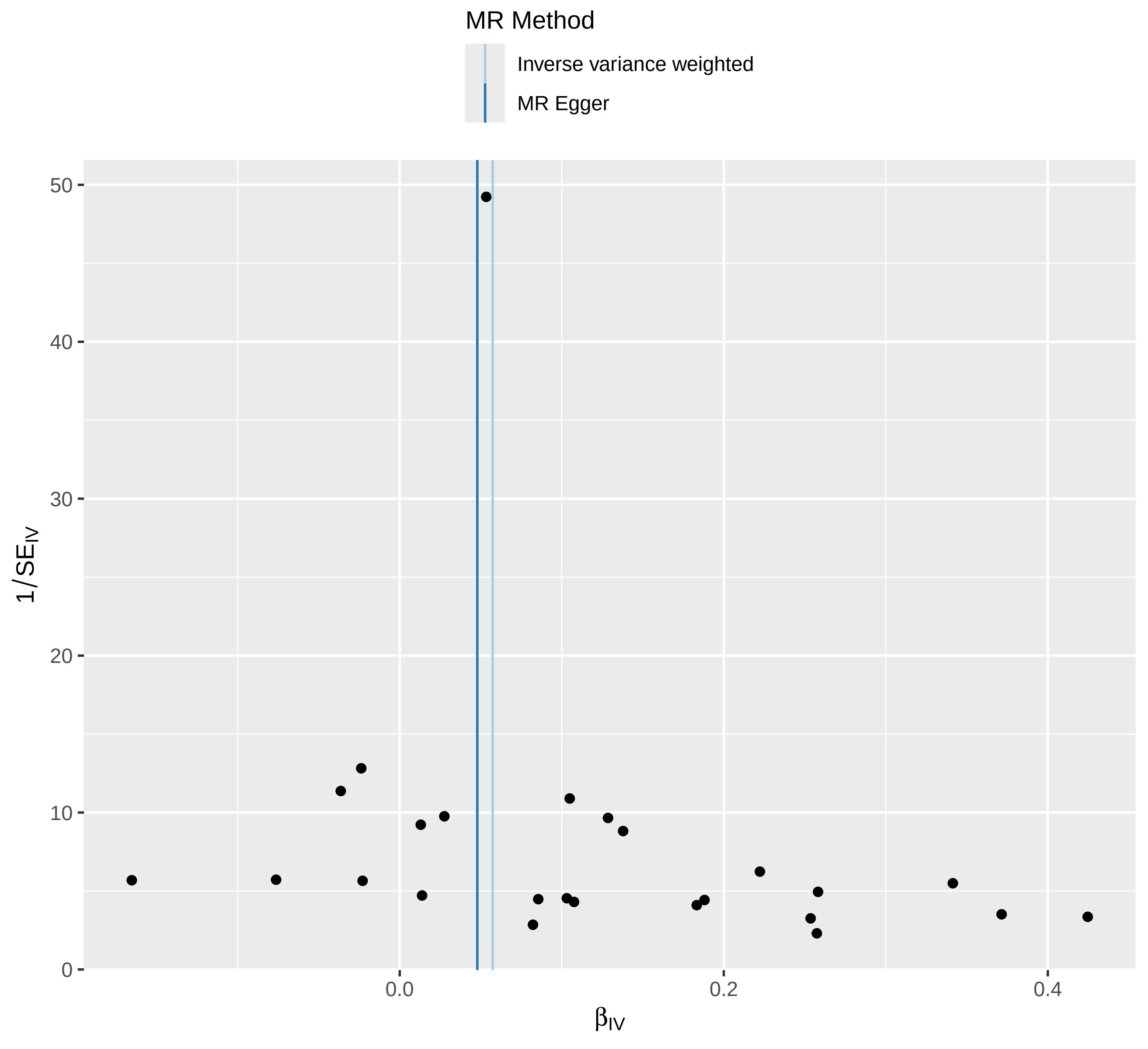
MR Method
Inverse variance weighted
MR Egger
50
40
30
20
10
0
1/SE_IV
0.0
0.2
0.4
β_IV

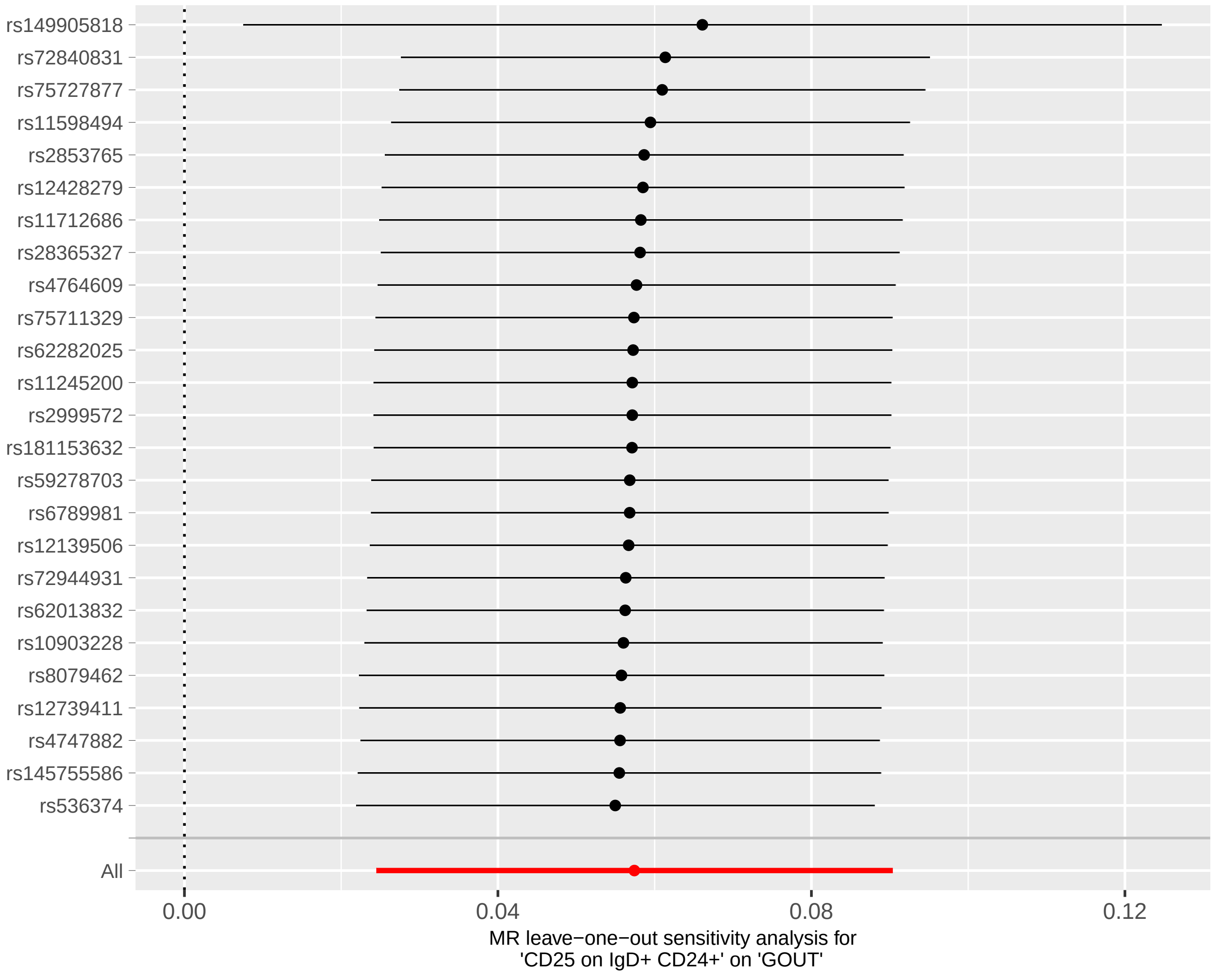
rs149905818
rs72840831
rs75727877
rs11598494
rs2853765
rs12428279
rs11712686
rs28365327
rs4764609
rs75711329
rs62282025
rs11245200
rs2999572
rs181153632
rs59278703
rs6789981
rs12139506
rs72944931
rs62013832
rs10903228
rs8079462
rs12739411
rs4747882
rs145755586
rs536374
All
0.00
0.04
0.08
0.12
MR leave−one−out sensitivity analysis for
'CD25 on IgD+ CD24+' on 'GOUT'

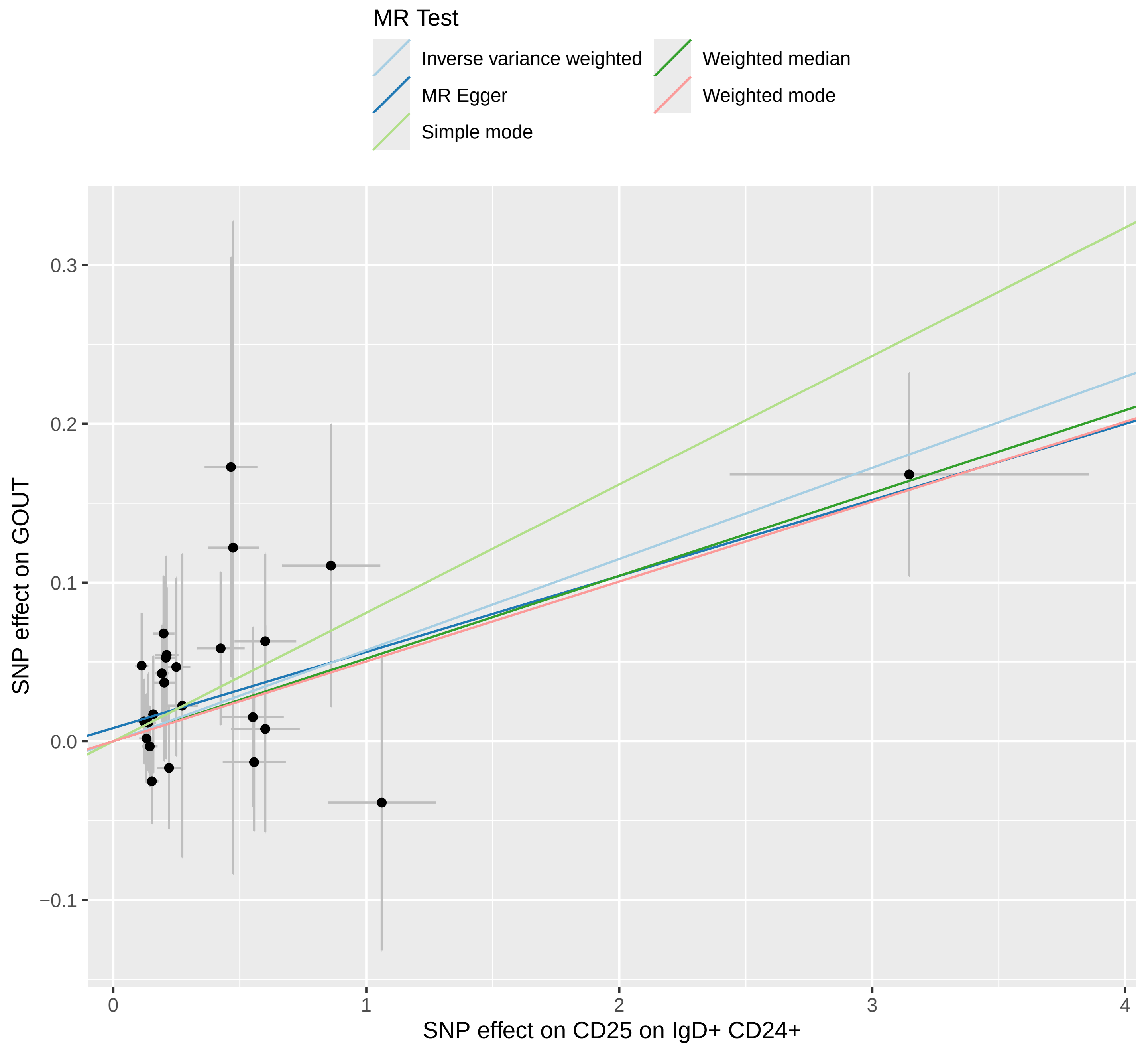
MR Test
Inverse variance weighted
MR Egger
Simple mode
Weighted median
Weighted mode
0.3
0.2
0.1
0.0
−0.1
0
1
2
3
4
SNP effect on GOUT
SNP effect on CD25 on IgD+ CD24+

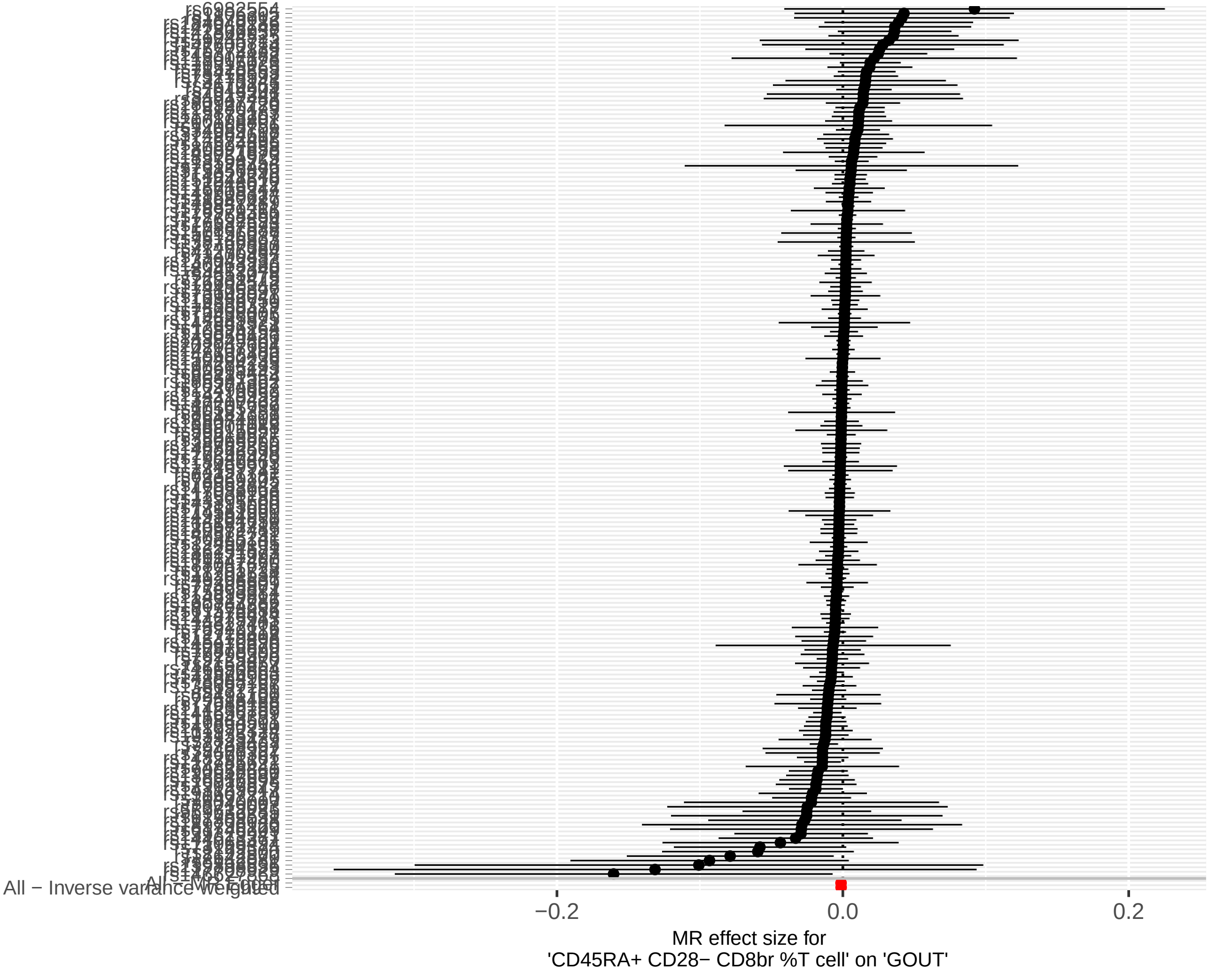

rs6982554
All − MR Egger
All − Inverse variance weighted
−0.2
0.0
0.2
MR effect size for
'CD45RA+ CD28− CD8br %T cell' on 'GOUT'

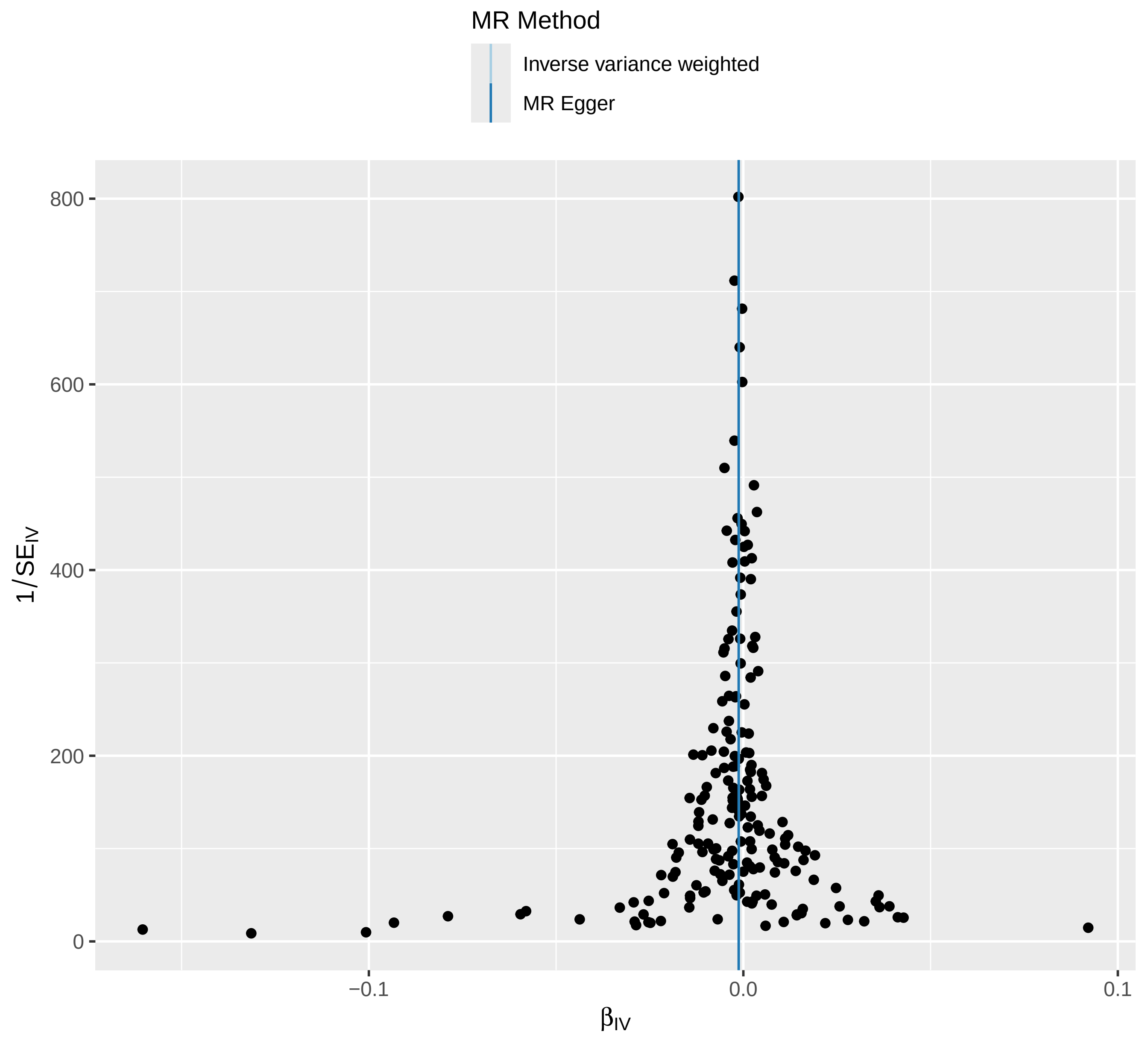
MR Method
Inverse variance weighted
MR Egger
1/SE_IV
800
600
400
200
0
−0.1
0.0
0.1
β_IV

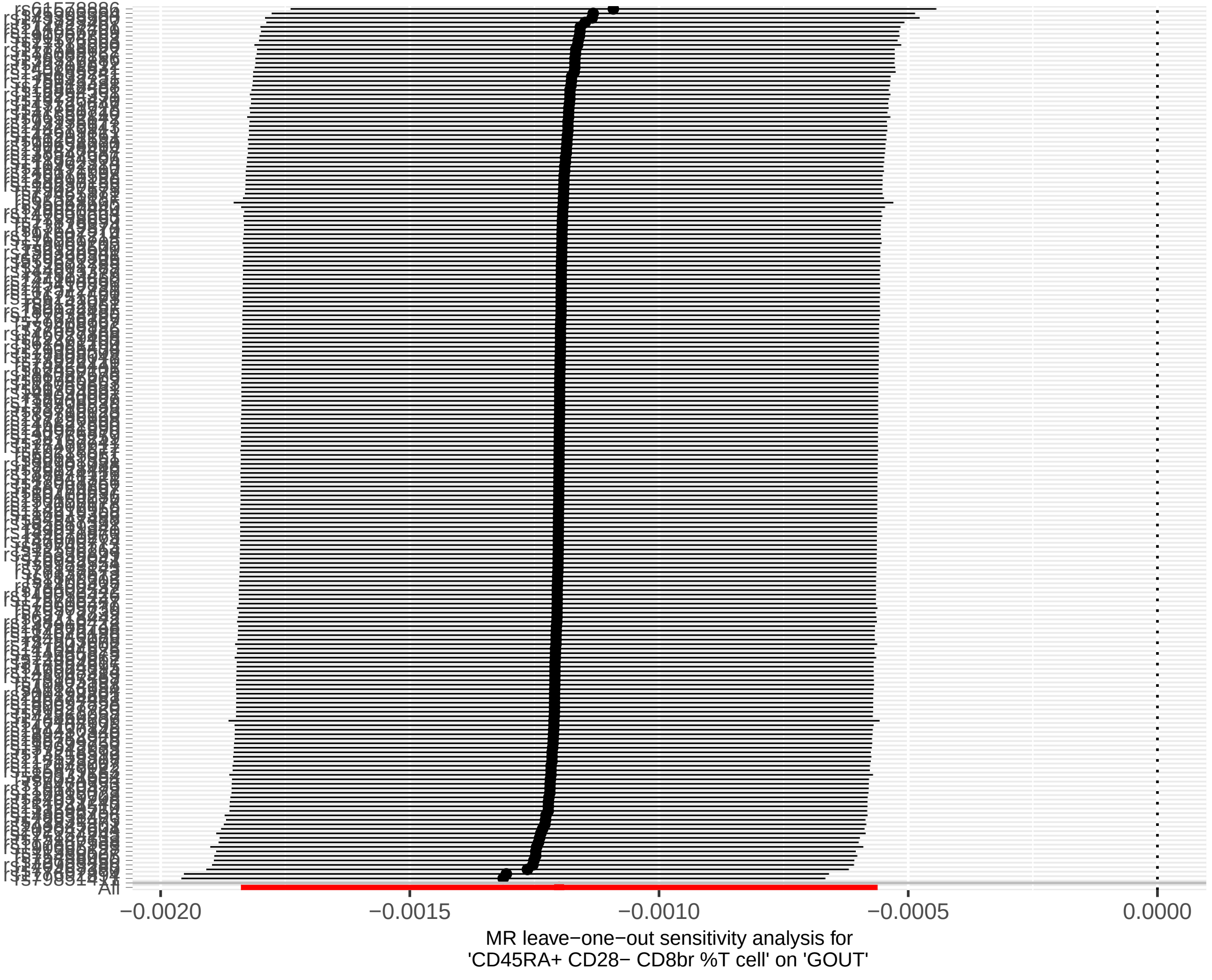

All
−0.0020
−0.0015
−0.0010
−0.0005
0.0000
MR leave−one−out sensitivity analysis for
'CD45RA+ CD28− CD8br %T cell' on 'GOUT'
